# Genome sequence of *Microbacterium foliorum* bacteriophage DumpQuist isolated from soil in Clarksville, Tennessee

**DOI:** 10.1128/MRA.00923-23

**Published:** 2023-11-22

**Authors:** Sergei A. Markov, Kehinde O. Olusoga, Patience O. Oni, Grace A. Henderson

**Affiliations:** 1 Biology Department, Austin Peay State University, Clarksville, Tennessee, USA; Portland State University, Portland, Oregon, USA

**Keywords:** bacteriophages, genome analysis, bacteriophage evolution

## Abstract

Bacteriophage DumpQuist was isolated from soil collected in Clarksville, TN, using the bacterium *Microbacterium foliorum*. Electron microscopy revealed that DumpQuist has a podovirus morphology. DumpQuist has a 53,924-bp genome that contains 54 predicted protein-coding genes and is most similar to phages in actinobacteriophage cluster EK1.

## ANNOUNCEMENT

**Fig 1:**
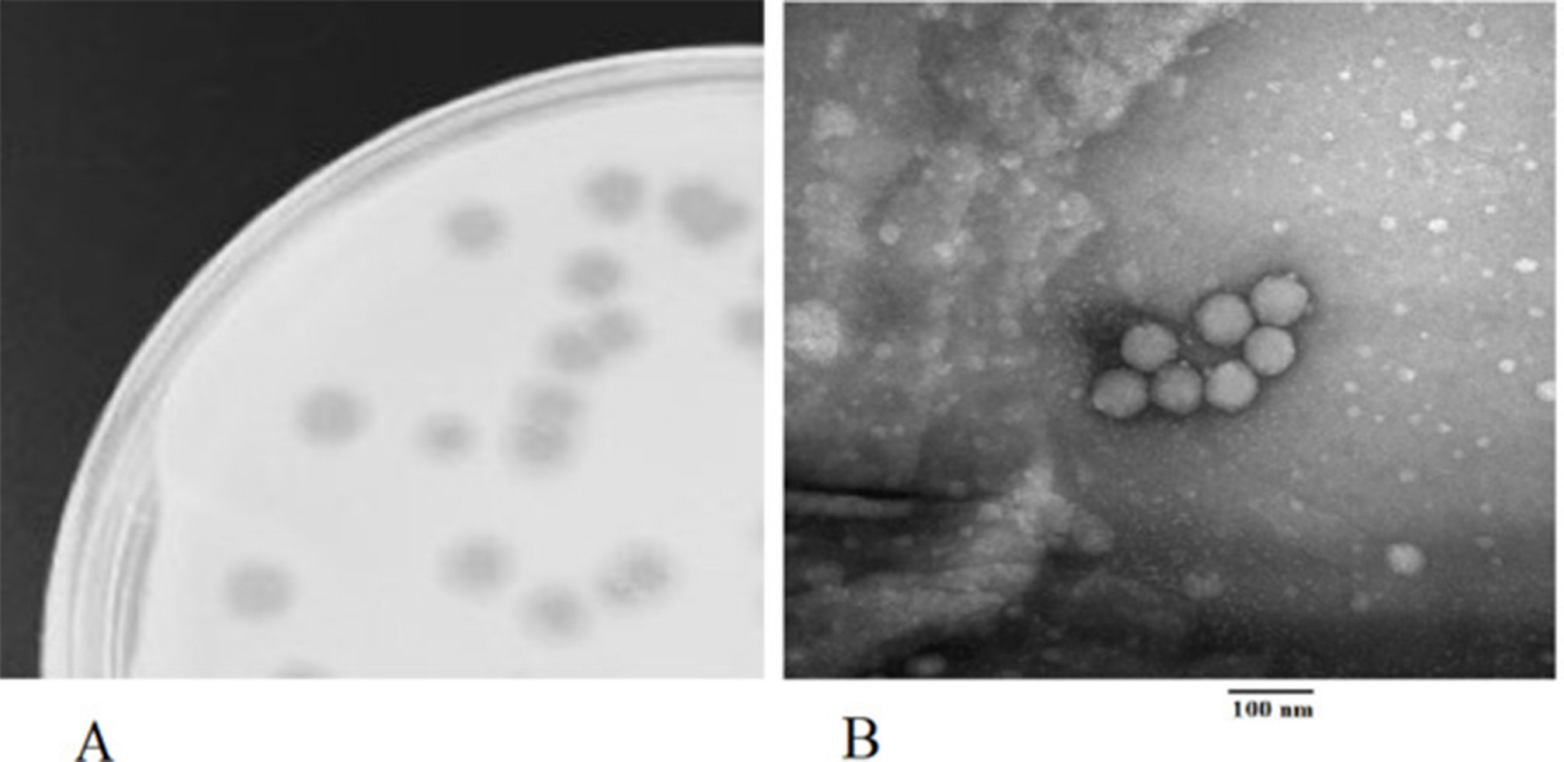
(**A**) Bacteriophage DumpQuist formed clear round plaques 4–5 mm in diameter. (**B**) Transmission electron microscopy photo of bacteriophage DumpQuist with a podovirus morphology with a short tail and an icosahedral capsid 52–54 nm in diameter (*n* = 7). The bacteriophage sample was stained using 1% uranyl acetate and imaged using a Hitachi H-7650 Transmission Electron Microscope (Tokyo, Japan) and an accelerating voltage of 100 kV.

We report here on the isolation and characterization of DumpQuist, a bacteriophage that infects *Microbacterium foliorum* NRRL B-24224, as part of our ongoing efforts to study the diversity of actinobacteriophages in Tennessee (1, 2). DumpQuist was isolated from a soil sample in Clarksville, TN (GPS coordinates: 36.5325 N, 87.35138 W) following the standard protocols outlined in a Phage Discovery Guide (https://seaphagesphagediscoveryguide.helpdocsonline.com/home). Briefly, a surface sample of wet soil was collected. The sample was then suspended in peptone-yeast calcium (PYCa) liquid medium for duration of 2 hours at 30°C. This suspension was then passed through a 0.22-µm-pore filter, and the filtrate was inoculated with *M. foliorum* and incubated with shaking at 250 rpm for 2 days at 30°C. Following this incubation, the culture was filtered, and the filtrate was plated in PYCa top agar with *M. foliorum*. After 2 days at 30°C, DumpQuist formed clear round plaques 4–5 mm in diameter (Fig. 1A). DumpQuist was purified using three rounds of plating. Transmission electron microscopy with negative staining (uranyl acetate, 1%) revealed that DumpQuist has a podovirus morphology with an icosahedral capsid and a short tail (Fig. 1B).

DNA was isolated from DumpQuist lysate using the Wizard DNA Clean-Up Kit (Promega, Madison, WI) and, subsequently, prepared for a sequencing using the Ultra II Library Kit (NEB, Ipswich, MA). The DNA was sequenced using a MiSeq instrument (v3 reagents) from Illumina (San Diego, CA) to yield 493,747 150-base single-end reads with 25-fold coverage of the genome. The raw reads were assembled using Newbler v.2.9 and checked for genomic termini and completeness using Consed v.29 as described by Russell (3). DumpQuist has a circularly permuted genome of 53,924 bp with a GC content of 60.1%. Based on the gene content similarity (GCS) of at least 35% to phages in the actinobacteriophage database, using the GCS tool (https://phagesdb.org/genecontent/), bacteriophage DumpQuist was placed in phage sub-cluster EK1, and it is most closely related to bacteriophage ArMaWen (96% nucleotide identity; 100% GCS) (4, 5).

The genome of DumpQuist was annotated using DNA Master v.5.23.6 with integrated Glimmer v.3.02 (6) and GeneMark v.2.5p (7), PECAAN (http://pecaan.kbrinsgd.org/), PhagesDB Blast (https://phagesdb.org/blastp/) (4), NCBI Blast (8), and HHPred v.3.2 (9), with default parameters for all programs. A total of 54 protein-coding genes were predicted, of which only 15 genes could be assigned functions.

Like other cluster EK phages, the rightmost two-thirds of genome contains predicted to encode genes for virion structure, assembly, and lysis; the leftmost third of the genome contains predicted to encode genes involved in DNA metabolism; and the center of the genome contains the largest actinobacteriophage gene, to date (5). In DumpQuist, this large gene (gene 31) is 4,481 bp. Consistent with other EK phages, no immunity repressor or integrase genes could be identified, suggesting that DumpQuist is likely a lytic phage (plaques were clear).

## Data Availability

GenBank and SRA accession numbers for DumpQuist are OR253898 and SRX20165761, respectively.
